# How civil society in India is marginalized. Civic space as relational process

**DOI:** 10.1080/17448689.2025.2515038

**Published:** 2025-06-23

**Authors:** Margit van Wessel, Rita Manchanda, Nandini Deo

**Affiliations:** a Strategic Communication Chair Group, Wageningen University & Research, Wageningen, The Netherlands; b Independent Researcher, New Delhi, India; c Department of Political Science, Lehigh University, Bethlehem, PA, USA

**Keywords:** Civic space, India, state-civil society relations, marginalization, international development, relational approaches

## Abstract

Restriction of civic space is widely understood as a condition that constrains the autonomous role of civil society organizations. However, this conceptualization is delimiting. This paper explores civic space as constituted in the dynamics between civil society organizations and state actors, contributing to an emergent shift to a more processual, relational and agential understanding of civic space, involving a redefining of civil society roles by state and civil society actors acting and reacting within their everyday work. We explore the case of India. Based on 36 interviews with state and civil society actors, the paper. shows how the state marginalizes civil society through three pathways: delegitimation, displacement and repurposing. A fourth pattern, however, qualifies this marginalization: political roles for civil society continue to be sought and found, depending on situations and the specific actors involved, based on their interpretations and political advantages at stake for them. The broader significance of these findings is, first, that everyday understanding and experience of civic space may prominently revolve around changes in civil society roles. Second, these changes in roles may best be understood at the level of concrete cases of relating and political contention, doing justice to the agency of the actors involved.

## Introduction

The role of many civil society organizations (CSOs) in development, peacebuilding, and humanitarian action is increasingly constrained (Biekart et al., 2023). Much of the literature on these restrictions treats them as external conditions that limit the autonomy of civil society. However, civic space is not just a condition – it is a relational process shaped by interactions between the state and CSOs. Existing research on state-civil society relations largely focuses on the dilemmas of co-optation and autonomy (Tadesse & Steen, 2019; Gutheil, 2023; Syal et al., 2021) or the instrumentalization of CSOs for state purposes (Toepler et al., 2020). Some studies take a more dynamic approach, analysing how restrictions are navigated (Fransen et al., 2021; Tadesse & Steen, 2019), resisted (Truong et al., 2024), or how differentiation processes reconfigure civic space (Falkenhain, 2020; Roggeband & Krizsán, 2021). Others highlight state-civil society interdependencies and the leverage CSOs can exercise within these relationships (Heiss, 2017; Dai & Spires, 2018).

However, what remains underexplored is how state and civil society actors themselves understand and shape their mutual relationships. This paper addresses this gap by examining how the everyday interactions between state actors and CSOs contribute to the construction of civic space in India. Using an interpretive approach (Bevir & Rhodes, 2015), we argue that civic space is not simply constricted but relationally reconfigured, with state-CSO interactions shaping roles, opportunities, and limitations. While prior research has described shrinking civic space as a function of state repression, our analysis emphasizes how role transformations occur through relational processes, rather than being merely imposed from above. Specifically, we identify three key mechanisms through which the Indian state reshapes civic space: delegitimation, displacement, and repurposing.

India provides a particularly compelling case for this analysis. While civic space has been legally constrained through regulatory restrictions and political pressures, civil society remains active, and state-CSO interactions persist. This study draws on in-depth interviews with both CSO representatives and (former) state officials who have directly participated in or observed these interactions at the federal and subnational level. Examining both perspectives allows us to capture not only how CSOs experience state-imposed constraints but also how state actors themselves justify, enforce, and interpret these restrictions. Our analysis reframes the study of civic space by demonstrating how state and civil society actors continually negotiate their roles in governance, policymaking, and advocacy. We show how CSOs seek to carve out roles for themselves, while the state simultaneously defines, limits, or denies these roles in specific policy engagements.

By approaching civic space as a relational process, we contribute to the growing body of literature that sees state-civil society relations as fluid and contested rather than simply hierarchical. Through the mechanisms of delegitimation, displacement, and repurposing, we illustrate how CSOs in India have been marginalized and redefined within a complex process in which the state holds the upper hand. State actors reproduce broader ideological narratives that justify restrictions on civil society while adapting these narratives to their specific institutional contexts. At the same time, CSOs continue to engage with the state, sometimes finding openings for collaboration despite structural constraints, and engagements can be deeply political in nature. These interactions confirm that civic space is neither wholly closed nor entirely autonomous but is instead shaped through ongoing negotiations.

The paper proceeds as follows. First, we present our theoretical framework, situating our approach within the broader literature on civic space and relational analysis. We then provide an overview of the Indian state-civil society context before outlining our methodological approach. The findings section illustrates the three mechanisms – delegitimation, displacement, and repurposing – through which the state reconfigures civil society roles. We conclude by discussing the broader implications of these findings for understanding civic space as a relational process.

## Theory and Context

According to the Civicus Monitor, in 2024 about 85% of the world’s population lived in societies with a repressed, obstructed or closed civic space (Civicus, 2024). Civicus defines civic space is defined as the respect in law, policy and practice for freedoms of association, peaceful assembly and expression and the extent to which the state protects these fundamental rights (Civicus, 2024). In recent years, research has investigated many dimensions of civic space constriction of civil society, developing and refining our understandings of what civic space constriction means. While we acknowledge that civil society is constituted by a wide range of organizations in India, some of which may not face constraints, in this paper we zoom in on national and regional non-governmental organizations (NGOs) and social movements that are involved in advocacy in the context of development, seeking to advance the rights, needs and interests of marginalized groups in India. The reason for this focus is such CSOs’ prominent relevance to inclusive development. Research on civil society in authoritarian regimes has stressed detrimental consequences of restriction of civic space for civil society's role of producing and sharing perspectives alternative to dominant state perspectives (Fransen et al., 2021; Toepler et al., 2020; Wischermann et al., 2018), essential to the advancement of rights, needs and interests of marginalized groups. Constrictions mainly pertain to freedoms (i.e., of expression, association, and assembly) and their enactment, especially through advocacy strategies, and especially in the public sphere, where regimes are openly challenged (Lewis, 2013). Restrictions mostly affect groups that are critical of government (Roggeband & Krizsán, 2021). However, any form of critique or form of contestation may be problematic, including in the context of constructive collaboration (Syal et al., 2021). Recent research reports that marginalization deepens with closing civic space, preventing broad civic engagement (Hossain & Oosterom, 2021).

Scholarship on civic space for civil society often centres on the space for organizations to act autonomously, representing societal views and interests, connecting citizens and state, and holding government to account. Research does increasingly addresses the ways CSOs navigate constrictions to manage their operational space. Common effects that research shows are CSOs responding by the stopping of operations, shifting from advocacy to service delivery, shifting topic and depoliticization of the advocacy (Fransen et al., 2021; Tadesse & Steen, 2019; Tadros, 2009; van der Borgh & Terwindt, 2014). Strategic navigation of restrictions also has been increasingly receiving attention. Researchers point to strategies like reframing into less-threatening language; shifting from national-level to local-level advocacy; shifting from agenda-setting advocacy to implementation; managing of visibility by using different platforms, supporting social movements behind the scenes; and building of trustful relations with state actors (Dai & Spires, 2018; Fransen et al., 2021; Gaventa et al., 2023; Tadesse & Steen, 2019).

Authoritarian and hybrid states in turn act strategically too, not just restricting but also instrumentalizing civil society, providing access and support to organizations that can boost government legitimacy, e.g., by providing services for citizens and confirming the validity and legitimacy of state ideology (Toepler et al., 2020). States may also permit and support (while delimiting foreign funding) CSOs to function based on roles and ideological fit, as shown by Fröhlich and Skokova (2020) for Russia and Liu and van de Walle (2020) for China. Such co-optation may be an attractive option for CSOs to advance their interest (Lorch, 2017) while it may also be a way to advance constituency needs and advance agendas shared with state agencies (Spires, 2011). Such literature sheds some light on interdependencies between state and civil society that provide some degree of CSO leverage on the state (Heiss, 2017; Spires, 2011). It also indicates the importance of exploring how CSO roles in restricted civic space can still take a variety of forms, which take shape through relational processes between the actors involved. This moves the discussion away from the starting point of autonomy, which posits the state as a constrictor, and towards the multiple ways in which state and civil society are ‘enmeshed together in a complex and multi-layered network of material transactions, personal connections and organized linkages’ (Lewis, 2013; cf. Chandhoke, 2001). Some research zooms in on this, showing how the perceptions, strategic considerations and relational work of the state and civil society actors are involved. Spires (2011) analyses how ‘illegal’ NGOs in China engage local officials to be able to do the work they want to do,

constructing a relationship that is symbiotic in that NGOs are looking to meet social needs, while government officials, especially at the local level, seek to make sure all ‘problems’ in their jurisdictions are dealt with in ways that do not attract unfavourable attention from their higher-ups (p. 12).

Tadesse and Steen (2019) similarly show how CSOs in Ethiopia navigate civic space by working on their individual position with state actors, for example through building strong relations with state actors who can help ease state control. Similarly, Syal et al. (2021) discuss development of trusting relations, within which some space for bringing in CSOs’ agendas may sometimes be created. Vu and Le (2023) analyse narratives and counternarratives in social media in Vietnam, from state and civil society, bringing out how this involves contestation of claims regarding the nature and legitimacy of civil society. Vértes et al. (2021) show how in Lebanon, civic space should be understood in terms of the intricate relations between state and civil society, in which sectarian politics have a key role to play. However, the ways in which individual actors’ interpretations come in, and implications of this for how civic space could be understood in the context of everyday reality, from both state and civil society, has hardly been studied.

Building on these foundations, this paper zooms in further on the relational work that is involved in giving shape to CSO roles: the mutual *sensemaking* and related everyday practices that build understandings and approaches for state and civil society. This is an ongoing collective process, carried out at multiple levels, by individual state agencies (more specifically their staff members) and CSOs. We offer a case study of India that, uncommonly, includes state actors next to CSOs. The article highlights both sides’ interpretations and actions as agents involved in reconstituting CSO roles in everyday practice. While embedding the study in the overall process of civic space constriction in India, we document and analyse civic space as understood and co-constructed, in interpretation and action, by actors directly involved, from an interpretive research approach (Bevir & Rhodes, 2015). This means that we try to capture how they see matters pertaining to state-CSO relations as the relevant reality here, while we situate their accounts in the context of the broader process of civic space constriction over past years. The reason for this is: while actors will be embedded in a social reality not of their making, how civic space develops will depend on how they relate to this reality and how they interpret situations, and engage and respond to each other on this basis. This is important because of three common characteristics of settings with constricted civic space. First, a state is not a single entity, but a connected set of levels and actors that approach civil society from their own perspectives, interests and agency. Second, in many contexts where civic space is constricted, the state is also porous, consisting of competing centres of authority that CSOs engage in diverse ways and in diverse capacities and relations, which we hardly take into account thus far (Lorch, 2017; van Wessel, 2023). Third, civil society is diverse and varieties of CSOs engage the state differently. We propose that this implies that different relations will be constructed by the actors involved, relating to the broader process of states’ civic space politics in diverse ways.

In India, public sector enterprises (PSE) contribute about 20% of the total national GDP and provide 40% of the wages paid in formal employment (Business Standard, 2022). Public sector enterprises include petroleum companies and textile producers. They are a hybrid form – both economic actors and government owned, therefore public agencies. These PSEs blur the boundary between the state and the market, much like Governmental Non-Governmental Organizations (GONGOs) blur the boundary between the state and civil society. The ideal typical forms of state, economy, civil society, and family that were imagined in the classic literature on civil society don’t quite capture the reality in India (Cohen & Arato, 1994).

During their decade-long rule (2004-2014), the Indian National Congress tried to advance a form of ‘inclusive neoliberalism’ – ‘market-oriented accumulation strategies coupled with social policy interventions that aim to protect poor and vulnerable groups from marginalization and dispossession’ (Nielsen & Nilsen, 2021, pp. 2–3). During the first decade of the 2000s, the government of the Congress-led coalition United Progressive Alliance held a secular orientation. It was on very friendly terms with some civil society groups through the National Advisory Council which reported directly to the head of the Congress party. During this time, India recognized the right to information, to food, to education, to work, and, for forest dwellers, the right to control their resources (Ruparelia, 2013).

Since the BJP came to power in 2014 leading a coalition titled the National Democratic Alliance (NDA) under the leadership of Narendra Modi, it has governed through what has been called ‘authoritarian populism’ (Nielsen & Nilsen, 2021). It has launched several populist welfare schemes, distributing material resources to populations as coming directly from the leader, rather than as part of a wider institutional system. Examples are sanitation, cooking gas provision and girls’ education. This government also took advantage of greater state capacity for surveillance, regulation, and investigation to target a range of civil society organizations, especially those seen as critical of the state (Khosla & Vaishnav, 2022). Criticism of economic policies and exclusionary majoritarian actions (and inactions) of the state have been the catalyst for investigation and suspension of registration of many CSOs, and punitive action against activists (Amnesty International, 2022). CSOs challenging the state are commonly delegitimized as ‘anti-national’ (Chacko, 2018). Academic freedom and freedom of the press have also been restricted (Chakrabarti et al., 2018). The Foreign Contribution Regulation Act (FCRA) is a major element in distinguishing between favoured and disfavoured CSOs. FCRA laws govern which organizations can receive donations from foreign organizations and individuals. Although first introduced in the 1970s, recent revisions to the rules have required CSOs to seek frequent renewals of their licenses, tightened accounting requirements and introduced new restrictions on funds (Chander, 2022).

A United Nations report classified Indian laws as unacceptable because of the risk that they will be used to silence CSOs whose views differed from those of the government (Kiai, 2016). However, as different authors have noted, civic space does not shrink for all civil society actors affected to the same degree or in the same way (Toepler et al., 2020). Organizations and activists associated with Hindu nationalism can operate with impunity and are even being legitimized and empowered through partnerships with the state (Vijayan, 2018).

And, while specific CSOs, journalists, activists and academics are countered by the state, thousands of CSOs continue to operate in India, including those with views and interests that are not in line with government agendas. Protest demonstrations continue to take place, despite being met with violence on several occasions. Moreover, many CSOs are involved with the state in collaborations (Syal et al., 2021). This varied and layered appearance of state-civil society relations begs the question how these differentiated relations are negotiated between the organizations and state agencies involved. We wish to emphasize that the state is not an unchanging monolith. Rather its relations with civil society are contingent on the ideology of the governing party, the leadership style of particular power holders, and the various levels and branches of governance.

## Methodology

To capture diversities in state-civil society interplay, data were collected in the capital city of New Delhi and the state of Jharkhand. Delhi was selected as the centre of Indian national government, with many national leading CSOs present and active in many domains. The young state of Jharkhand (created in 2000) was selected as an arena to see how state and CSOs interact at the grassroots and subnational level. At the time when the interviews were done, the BJP was ruling in the Centre and the state. Given India’s diversity, the inclusion of only the state of Jharkhand and the capital limits representativeness. However, we can conceptualize this twin focus as a case of state-CSO relations around nationally administered development schemes widely involving CSOs across India. Jharkhand, being a case of low social development, a high percentage of disadvantaged rural communities, and strong presence of CSOs active in Jharkhand and beyond, offers then a site where themes and tensions around CSO involvement in development can be expected to present themselves clearly in India more broadly. This leads us to expect that our findings may apply to other settings in India where similar forms of marginality, schemes and CSO roles can be identified.

This paper draws upon 36 semi-structured interviews with both state and civil society actors at national and state/sub-state levels, carried out in 2018 and 2019. State actors included state Ministers, national, state and district-level senior and mid-level civil servants, representatives of state bodies and lateral entry ‘consultants’ in state agencies. Also staff of hybrid ‘government-organized non-governmental organizations’ (GONGOs) were included. In view of our focus on state-CSO engagement in responding to the development needs and rights of poor marginalized rural communities, we selected interviewers connected with the state who were responsible for rural development, social welfare and justice, indigenous rights, food and public distribution, agriculture, forest rights and women’s empowerment (Table 1).

**Table 1.** Interviewees.

State-level elected officials and civil servants – Jharkhand	4
Central government civil servants – Delhi	5
Ex-Central government civil servants – Delhi	4
State-level and + sub-state level CSO leaders – Jharkhand	9
National-level CSO leaders – Delhi	10
GONGO staff – Jharkhand	2
Scholar/activist – Delhi	1
Niti Aayog consultant – Delhi	1
**Total**	36

A few officials were retired national civil servants who were recommended by trusted CSO interlocutors as being perceptive and communicative. One author’s years as a professional journalist helped to facilitate access to state circles, as did our project collaboration with the prestigious Indian Institute of Technology Delhi. On the civil society front, the co-author’s previous experience of professional interface with CSOs enabled ease of access. Respondents were directors and senior level staff of eminent national development CSOs with a footprint in Jharkhand; national-level NGOs with a record of engagement with government; state chapters of NGOs working cooperatively with the state on delivery of welfare schemes and advocacy; state-level CSOs juggling multiple identities with cooperative and confrontational relations; Food Rights, Forest Rights and Right to Work campaigners, local NGOs, including women’s groups, working with and working the state; networks and social movement alliances. With this sample of national and state-level state and civil society actors, we have obtained a spread of views in terms of thematic focus and context. About 15 interviews were recorded (with permission), spanning both state and CSO actors and these were then transcribed. In the case of interviewees reluctant to be recorded, extensive notes were taken and then promptly written out in full as much as possible. Fieldwork (2019) was supplemented with document analysis (official and CSO websites, grey literature and media reports). Qualitative analysis of the data was grounded in deductive and inductive coding, from a grounded theory approach (Strauss & Corbin, 1994) to capture the mutual perceptions, (dis)engagements and narratives by which state and civil society actors make mutual sense of their relations and define what state and civil society can be to each other in a specific context. This coding was done manually. First, the broad themes of delegitimation, displacement and repurposing were identified through open coding, allowing for understandings to emerge from the data. After key themes became evident, focused coding was used to refine these categories, identifying patterns within and between them, and learning about the interplay between state and CSOs. To protect identities, no names of individuals and organizations are disclosed in this article. Our data are about six years old and the election of 2024 reduced the parliamentary dominance of the ruling BJP government. As yet there has not been a major realignment in how the state interacts with civil society actors, so we believe our findings continue to be relevant to theorizing how civil society and the state make sense of each other.

## Findings: Marginalization and Collaboration

The most prominent patterns are marginalization through three pathways: delegitimation, displacement and repurposing. Together these pathways illustrate the everyday reality of civic space not as one of simple constriction, but of minimizing CSO roles in society – a significant difference that highlights the experience of transformed relations: moving apart, exclusion and loss of relevance for CSOs. A fourth pattern, however, qualifies this marginalization in two ways. First, individual state actors continue to see value in working with CSOs. Second, political considerations remain a foundation for collaboration between state and civil society, pursued on both sides, depending on situations and the specific actors involved. This undermines the seemingly totalizing quality of marginalizing state discourse and practices. Below we discuss these developments as relational processes, connecting state and CSO perspectives and experiences.

### Marginalization Through Delegitimation

Interviewees associated with the government discursively delegitimated CSOs by constructing them as suspect and therefore no longer worthy of collaboration. Advocacy, and especially campaigns for social justice, were problematized as ‘political’ with this descriptor carrying the negative connotation of working against the government, and being anti-national and anti-development. Politicians accused CSOs of holding a partian bias rather than being ‘neutral’ as they should be and thereby sacrificing the peoples’ interest. A state Minister who had earlier welcomed activists as partners was particularly critical. ‘CSOs are not open-minded but have a fault-finding approach, and work in league with the political opposition’. Smarting at reports of hunger deaths and deficiencies in the implementation of the Food Security Act, the Minister was scathing about academic activists, ‘outsiders’ who worked collaboratively with the previous government, but now, they ‘join the political opposition and take out a protest rally. How can we believe they are social activists. … .They rush to Twitter and Facebook to get international exposure. Instead of dialoguing with us, they parachute to Delhi and create a controversy there’. CSOs are anxious, lest they be viewed as aligned with the political opposition, illustrated by young activists who spoke of their hesitation to reach out to potential local allies if they happen to belong to an opposition party. They perceive swift official displeasure, if, for example a CSO staff member was spotted campaigning for an opposition candidate in elections. Reflecting on the shifting dynamics of what state officials considered as acceptable CSO roles and action, a convenor of a major rights campaign said, ‘Previously, a *dharna* (public protest) would open the way to dialogue; a public hearing drew political leaders from across parties, and within the then Planning Commission there were sympathetic members who could be accessed’. This is no longer seen as a pathway to productive engagement.

Government interviewees also questioned the agenda of minority faith-based CSOs, singling these out as suspect development actors. Official respondents were outspoken against the work of Christian organizations – ‘because their agenda is to convert tribal^1^ people, not development’. Although Christian missionaries working with indigenous communities in India have been entrenched in social welfare activism, the unquestioned assumption was that their real agenda was religious conversion (cf. Bauman & Ponniah, 2017, p. 68). This is in line with national policy. Already, under FCRA rules (2011) CSO office bearers need to declare that they are not involved in religious conversion. Reportedly, 70% of the religious CSOs whose FCRA registrations were ‘deemed to have ceased’ were directly or indirectly related to Christianity (Rao, 2022). The BJP government in Jharkhand has also initiated criminal investigations into the financial dealings of 106 Christian-affiliated CSOs. Interviews illustrate the broad and invasive suspicion against minority faith-based organizations as part of everyday reality. For instance, a mid-level state official working in a forest district said,

The churches were getting a lot of funding from Germany and the UK. There was a lack of information about what they were doing. No transparency in their funding. There was strong evidence that they were supporting extremist groups.

A programme supervisor posted in a forest district alleged that substantial foreign funds for the Church social welfare activities were being diverted to supporting Left Wing Extremist/Naxal activities. A state-level minister added that meetings of activists who were critical of government policies towards the poor, ‘take place in Christian institutions’. This referred to the last-minute cancellation of the venue for holding the bi-annual meeting of the Right to Food Campaign in Jharkhand. Eventually it was held in premises provided by the Missionaries of Charity, a Catholic network set up by Mother Theresa, which is not a political group.

CSO respondents also spoke of the discriminatory singling out of minority faith-based organizations in the government’s CSO watchlists, and the way in which suspicion can define relations. An example is Jharkhand’s controversial incident in district Khunti in 2018, in which four women who were associated with a Christian NGO and were performing an anti-trafficking skit were abducted and raped. Attempts at an independent inquiry into the incident were blocked, according to a women’s legal aid group and they claimed that the incident was manipulated to undermine the Missions’ schools and hospitals (see also Sinha, 2018).

Another common narrative was of CSOs as ‘non-profits only in name, pursuing self-interest’ as a state Minister put it. As evidence she alluded to a call for a social impact assessment, for which 53 CSOs sent in such unrealistically low bids that ‘it was clear they were only interested in getting empanelled to make money’. CSO feedback was not useful, better to do the verification ourselves, she claimed. A central government official who recognized the need to involve CSOs for outreach and expertise in implementing an all-India anti-addiction survey for determining disbursement of central government monies, confided that she had hesitated because, ‘so many NGOs can be frauds. *Paise banate hai*!’ (They make money). Relatedly, CSO effectiveness too was questioned by officials. ‘Earlier, we worked directly with hundreds of NGOs! … . [They] failed to produce results. Now, it is government which itself is doing the maximum amount of work on *gramin vikas* (rural development)’. And: ‘Vast majority of CSOs have no capacity to deliver’. Echoing the opinion of several public servants, an official defensively asserted ‘you hold us [government] accountable, what about CSOs? They’ve been working for years, where is the promised social change?’ By claiming CSOs are politically partisan, communal, and/or corrupt this discourse delegitimizes them.

### Marginalization Through Displacement

As indicated above, government officials spoke of ending collaborations with CSOs. For CSOs, this withdrawal is paramount in their experience of changing relations after 2015. As the director of a well-known CSO, once a privileged insider in government-CSO policy interactions observed, ‘the signal from the Prime Minister’s Office was clear, ‘do not engage’ with NGOs’. As evidence of that change, while previously there would be CSO standard bearers at the consultative table of *NITI Aayog* (the government´s apex public policy think tank), today there are techno-managerial consultancy firms and corporate social bodies. Some reporting on the Indian state’s increasing reliance on consultancy firms and technocratic perspectives is emerging (Sajjanhar, 2022).

Many CSOs viewed their relegation to the periphery with a mix of resentment and anxiety. A habitual CSO invitee to government committees explained that the state’s new development partners are corporations – Piramal Education Foundation, HCL, Gates Foundation and transnational consultancy firms like McKinsey, Ernst and Young, PwC and Boston Consultancy Group. CSOs find themselves at the losing end in a new competition for influence. *NITI Aayog’s* web portal *Darpan* is the gateway for registered CSOs to secure government funds. Contracts are awarded through a competitive tender process but given the scale and monies involved, even CSOs known for their core competence, for instance on women’s issues, have lost out to PwC, they lamented. In this new competition, the terms have changed, with the critical role of CSOs losing its relevance.

According to CSO interviewees, the government’s new development partners don’t provide critical perspectives. For instance, as a CSO director observed, the new ‘development partners are not interested in bringing you critical feedback from the ground’. Officials and CSOs understand and work with the reality that the government has pushed back at what it perceived as CSO encroachment into their territory of social governance. While none of the CSOs addressed allegations of corruption or claims that their activities were suspect, several CSO contested the image of ineffectiveness. They grumbled that they had set up the ecosystem of self-help groups (SHGs) that had been appropriated by State Livelihood Promotion Societies, (SLPS) which are civil-society like agencies set up by the state (i.e., ‘GONGOs’, see also Hasmath et al., 2019) without acknowledging their contributions. CSOs also recognized that their relevance was contested and denied, with new actors coming into favour. These GONGOs are official imitations of CSOs and are being promoted by government agencies as the implementing bodies for the Ministry of Rural Development’s National Rural Livelihood Mission (NRLM). The NRLM programme has monopoly control over rural development and aims to benefit some 85% of the country’s rural poor. The SLPS are the primary vehicles for implementation of the NRLM and are funded by the Ministry of Rural Development. Through microfinance they seek to organize and mobilize the poor, especially women. According to the Ministry, by 2021 more than 81 million women from poor and vulnerable communities have been mobilized into some 7.3 million SHGs (Ministry of Rural Development, 2021). Previously, hundreds of CSOs were engaged in mobilizing community-based collectives, especially SHGs. Several CSO interviewees decried their displacement in this regard. In relation to state agencies' recapture of SHGs, the director of a Jharkhand-based CSO spoke of the government’s ‘engulfing’ civil society mobilized SHGs. ‘We created a lot of SHGs, but they all got merged in the JSLPS programme. So it’s like we worked for something, and someone else took the benefit’.

One senior government official pointed out the incorporation of the social audit model developed by a CSO in the Societies’ operational structure as an example of collaboration. But a government consultant had another reading:

These Societies have appropriated the philosophy of community-based organisations, but work like a government scheme and programme. [Societies] are trying to capture or appropriate the spirit of a people’s movement, poaching CSO workers, but bureaucratizing the whole process, making the SLPS a government department. Even the independent audit unit is housed within the JSLPS building.

A convener of a women farmers’ platform singled out the difference, underlining a pattern of appropriation and transformation. ‘The feminist culture of SHG federations of fostering trust and solidarity has been taken over by a culture of corporatization’. Two senior civil society leaders added that SLPS have been expected to produce beneficiaries who would be ‘loyal’ political cadres of the regime and uncritically carry forward government flagship schemes

Contributing to the displacement of CSOs in their critical role is also the implementation of the Companies Act of 2013, which makes it mandatory for large companies to spend part of their revenue on charity – what is called Corporate Social Responsibility. For some CSOs this can form an alternative funding source. The law generates at least 225 million USD (around Rs 18,5 billion) annually. However, some CSOs complain of corporate philanthropy hoarded by the social foundations of these businesses, which may be an extension of the corporation’s core competence areas – even though this is contrary to the spirit of the law. ‘Their social service lines serve to legitimize or further their core competencies or to ingratiate them [the corporations] with the government’, an activist said. CSR partnerships are often driven by corporate priorities with CSOs treated as implementing agencies, thus undermining the credibility of the CSOs themselves (Deo, 2019). Corporate and non-profit partnerships are thus not relationships of equals, but can further marginalization for CSOs (Deo, 2024).

### Marginalization Through Repurposing

The state has sought to redefine CSO roles, not only controlling but also repurposing them by relegating them to ‘helping hands’. While the continued acceptance of CSOs in service delivery roles has been widely reported (see e.g., Toepler et al., 2020), we show how this can involve processes of recreating relations on new terms. With the new BJP-led government in 2014, the disciplinary powers of the FCRA amendments of 2010 came to be wielded extensively. The rules broadly delegitimize political activity by CSOs, problematizing an organization as political when they, for example, seek to advance political interests of oppressed castes or workers, or use strategies like *bandh* (strike), *hartal (closure), rasta roko* (road blockade), *rail roko* (rail blockade) or *jail bharo* (voluntary mass arrests) in support of public causes (see Ministry of Home Affairs, 2011). CSO directors referred to the punitive costs of being singled out for taking ‘political action’. For instance, an ethno-medicine expert keen to get the Jharkhand Ministry of Health to collaborate in developing an indigenous medicines programme, interpreted the Chief Minister’s peremptory dismissal of the project proposal as related to his negative comment on her participation in an anti-dam protest rally, ‘You didn’t allow the Koel Karo dam to be built’. Recently, a director of a women’s rights CSO participated in a rally in Ranchi on displacement, organized by the National Alliance of People’s Movement. Reportedly, that evening he got a call from the Central Bureau of Investigation. According to a colleague, ‘it was just a general enquiry, but it was made clear, he was under watch’.

The government says it intends to allow only ‘genuine’ and ‘authentic’ CSOs working for the ‘welfare of the society’. This is cited as the rationale for the FCRA Amendment Bill, 2020 (Ministry of Law and Justice, 2020). ‘Authentic’ CSOs is a phrase echoed by state politicians and local officials as they differentiate credible CSOs from those ‘making money’ or serving duplicitous agendas. Such CSOs act as ‘helping hands’ of government delivering welfare services. This relegates other CSOs roles to illegitimacy. According to the Ministry of Home Affairs, 16,754 CSOs have been barred from accessing foreign funding since 2014 (Chander, 2022). Activists interviewed for this study also referred to lists of CSOs prepared by the government in 2015 based on inputs from the Intelligence Bureau (2014), claiming they were implicated in suspect activities such as supporting left-wing extremists, converting indigenous people to Christianity, and associating with sectarian organizations, such as the Students Islamic Movement of India.

Laying down boundaries for legitimate CSO action, a programme manager in a GONGO described, ‘the submission of an MoU [Memorandum of Understanding]’, as an acceptable activity, but ‘not a *dharna* (public protest)’. A veteran CSO director with wide community reach iterated,

We [CSOs] are expected not to make things difficult for the government; not to encourage or facilitate the communities we work with to ask difficult questions; we are expected to reach government schemes and programmes out to the people.

A CSO in Jharkhand and another in Rajasthan, suffered the consequences of being associated with mobilizing community-based SHGs that gave critical feedback on the functioning of the Prime Minister’s flagship schemes – *Swachh Bharat* (clean India), Pradhan Mantri *Awas Yojna* (affordable housing), *Ujwala* (gas connection) and the continuing MGNREGA (right to work) programmes. CSO respondents felt that any criticism of the implementation of these schemes was taken as detracting from the Prime Minister’s highly publicized achievements. In the government’s design, the SHG ecosystem was the primary vehicle for implementing the Prime Minister’s flagship schemes. The partnering CSOs had set out to collaborate with the government. But the process of mobilizing women’s economic capability and leadership led to growing consciousness about rights and justice, and eventually a confrontation between government and CSOs (Manchanda, 2023). As a mid-level CSO professional working at the grassroots decried, ‘the community that the CSO works with gets so identified that even a spontaneous community activity gets associated with it.’ And there were hostile inquiries about which staff member was involved. The government’s displeasure is made clear, ‘they [government officials] don’t know how to respond to people’s democratic assertion’, a CSO director said. The CSOs are held responsible for the accountability demands of people in their areas of outreach.

## Collaboration Through Situationally Defined Civic Space

While the repurposing of CSOs from advocacy to service delivery restructures relations and roles between the state and CSOs, interviewees from both state and civil society also saw the scope for mutual engagement as situational, and as developed in interplay.

### CSO legitimacy as a Valued Resource

Interviewees associated with the government indicated variation in specific actors’ relations. Reflecting on what he saw as the arrogance of the country’s elite public servants and their ‘resentment’ of CSO ‘encroachment’ into their territory of social governance, a former public servant spoke of his colleagues being unjustly ‘dismissive’ of the contributions of CSOs. A consultant assigned to NITI Aayog was struck by the persistent refusal of the administration to acknowledge the significant contribution of CSOs in the mobilization of women’s SHGs, now appropriated by government-directed GONGOs. Here we see that relations can develop in different ways depending on the situation and the dynamics between the actors involved. An official with a history of partnering with CSOs in developing India’s Andaman Islands said she had ‘to overcome the resistance of colleagues and subordinates’ who distrusted CSOs. But she also observed that within the administrative system, ‘there will always be pragmatic officers, especially community block officials because they are at the ground level’, who will partner with CSOs. Several CSO actors concurred that confrontation often was with central and state bureaucracy at headquarters, not local officials needing mutual understanding with local CSOs to deliver on government-set targets (cf. Spires, 2011). Politicians too, were not viewed as the problem despite the competition for credit. ‘If the politician knows a CSO has real influence in the area, he will not go against you. More likely say ‘put in a good word for him’. Politicians need political legitimacy. Bureaucrats don’t’.

Resisting narratives of state withdrawal due to CSO corruption, a senior official observed, ‘there are some complaints but not much; you get similar complaints about the government sector’ and a former civil servant explained that ‘officials would not want to work with CSOs that have a culture of accountability. There is no difficulty in lining up some “friendly” CSOs to do implementation. It is part of the culture of “cronyism” in government’. A grassroots civil society respondent in turn expressed reluctance to work with the state government given the entrenched culture of bribery and other malpractice on the state’s side. Several heads of government departments also said that they looked to CSOs to counter such anticipated malpractices, especially capture by local politicians of livelihood schemes that involves brokering people’s entitlements on the basis of a payment.

### Political considerations

Activists also indicated that the scope and extent of the roles of civil society in political action depends on their strong community roots. As scholars Glasius and Ishkanian (2015) analysed in another context, community rootedness can provide financial independence and a capacity to leverage the fractured field of power as a political actor enmeshed in local political relations and alliances. Activists toggling between the bounded discipline of an FCRA-registered CSO and their involvement in a rights-based people's struggle, spoke confidently of their capacity to counter government action against them.

I’m not scared of the government’s hostility, because stoppage of funds will not close our local work as long as we are supported by the community … Our movement is strong with capacity to intervene politically and even make a difference electorally as we demonstrate by putting up an activist candidate in the assembly elections who defeated the then ruling political party.

Government officials in turn suggested a mutual awareness and even tolerance among collaborating stakeholders – government, donors, CSO and communities – to relationally work out what each is looking to the other for in a specific context. For example, the head of a government department responsible for reaching out to remote indigenous communities was frank about the need to partner with CSOs and was quick to add, ‘so long as they (CSOs) have capacity to mobilise, they will be valued by the politically powerful in performing politics’. One CSO that has such power was initially told – ‘focus on livelihoods, leave the social mobilizing to us’. Its contracts were suspended. Subsequently, top officials reached out to the CSO to mediate a brewing state-people confrontation. This shows how CSO capacity for grassroots mobilization can be both a threat *and* an opportunity in the eyes of elected state officials. The mutual need for political legitimacy creates scope for advocacy and political civic action with their own respective constituents, thus requiring the state and CSOs to flexibly navigate how they relate.

Political roles are thus not fully undermined, but can emerge in situations depending on the potential for mutual advantage. Another illustration of this is that of the aforementioned Right to Food campaign, wanting to organize its biannual meeting 2016–2017. Activists spoke of the many hurdles officials raised to block the meeting at first, but also pointed out that eventually it became the forum for the Chief Guest, the Minister, to announce supplementary nutrient funds to be managed by a government-directed organization.

CSOs capitalize on the opportunities of working in a fractured field of power, linking up with the state in varied ways, through the grounded knowledge and relations they have in their respective contexts. An activist associated with the Forest Rights campaign described working as a political actor enmeshed in political relations to counter central agencies’ punitive action, and later being invited as a state nominee to speak for the vulnerable in a government policy committee. A CSO whose primary identity was left, progressive, and autonomous and often in confrontation with the state, took care to collaborate with a state agency on a small project on migration. State funding was minuscule, but the token collaboration was strategic, to maintain links with executive power and policy. To maintain advocacy roles, CSOs have also made strategic use of the diversity of civic formations in the civil society sphere and the diversity of the interpretation of CSO roles (Singh & Behar, 2017) to juggle multiple identities and build bridges across differently oriented platforms. Confronted with a restrictive civic environment, and one particularly intolerant of any ‘political’ role of CSOs, we observed a notable CSO praxis of strategic stealth to create space for more radical civic action by juggling between two or more platforms. One can be a registered as a ‘development’ CSO bound up within the disciplined logic of FCRA; the other, an independent non-registered self-financing ‘political’ platform working for structural change. Many FCRA-registered CSOs work collaboratively with the government, while also partnering with alliances, membership-based networks, labour unions and social movements that occupy more contentious and adversarial spaces. Such twin platforms and bridging alliances were spoken of as commonplace. For example, a registered research and training institute was twinned with a non-registered peoples’ movement for justice and rights; an ethno-medicine trust was linked with two social advocacy platforms; a funded programme for social action had floated an activist space in Delhi linked with social movements and alliances which take adversarial stances. In this way, CSOs could maintain legitimacy with the state while still being involved in action that may be disruptive in the eyes of government actors. Whereas in some cases CSO professionals were at pains to keep the two identities separate, in other cases, the distinction is a fiction. Certain CSO websites even acknowledged the dual platforms. Others practiced stealth or management of visibility, limiting involvement to facilitating venue, travel and political outreach to overcome bureaucratic hurdles of access. Surprisingly, certain officials expressed awareness of the co-existence of dual platforms. Asked whether it complicated possibilities of such CSOs working with the government, especially when the radical platform engages in agitation politics such as a *dharna* (public protest), ‘*dharna* is not a criminal activity’, the official said. CSO partnered with radical platforms drew upon the protective support of their rootedness in local communities and their capacity to be a political actor in the local field of power.

## Conclusion: A Qualified Marginalization in Action

While most research on civic space centres on state constrictions and CSO navigation, the interpretive approach taken in this study brings out how the actors involved partake in the construction of meanings concerning civic space and give shape to these relationships in everyday practice. While this was an exploratory study of limited scope, useful for generating theory rather than testing it, we believe our findings offer significant insights deserving of further exploration to understand how developments in civic space are experienced and related to in society.

Two main insights stand out for us. One, this study foregrounds *marginalization* as a prominent dimension of civic space developments in India in recent years. The message to CSOs now is ‘you are nothing to us now’ as much as ‘you are bad’, and this is also part of CSOs’ experience. The broader significance of this finding is that everyday understanding and experience of civic space, as an issue, may prominently revolve around changes in roles – from being valued by the state, to being something else – in this case: sidelined and repurposed, with some qualifications depending on the actors at hand.

Second, marginalization is not merely through generalized, overall rejection and restriction. The denial of a legitimate role is enacted within concrete cases of relating and political contention between state and civil society, pushing back against CSOs that are claiming roles. State agencies draw on state discourse, reproducing and applying it in their own contexts. This happens within specific cases of contention, with diverse intents and outcomes depending on the way state actors involved approach the situation. Personal relations continue to make a difference. This has been identified before in navigation of civic space (Dai & Spires, 2018; Fransen et al., 2021; Gaventa et al., 2023; Tadesse & Steen, 2019). However, our study also indicates that both government and civil society actors sometimes do value political roles for civil society, depending on situations and the specific actors involved. Mutual political advantage remains a foundation for collaboration between state and civil society that thus has continued to be pursued in varied ways, recognizing and putting to work the political role of civil society that is otherwise so often delegitimized and denied. To understand how CSOs can strategically engage civic space conditions, we need to look beyond strategic navigation, and include mutual political advantage as a novel entry point for research as well as strategy in practice.

## Note

1 While in this paper we speak of indigenous groups, in India the terms ‘tribal’ and ‘tribals’ are commonly used to refer to indigenous groups in government policies, legal frameworks, academic discourse and NGOs parlance. The term *Adivasi* is often used by activists and community members referring to identity and rights of indigenous communities, emphasizing rootedness in indigenous setting and historical connection to the land, often associated with struggles for recognition and land and forest rights.
